# Alphavirus Replicon DNA Expressing HIV Antigens Is an Excellent Prime for Boosting with Recombinant Modified Vaccinia Ankara (MVA) or with HIV gp140 Protein Antigen

**DOI:** 10.1371/journal.pone.0117042

**Published:** 2015-02-02

**Authors:** Maria L. Knudsen, Karl Ljungberg, Roger Tatoud, Jonathan Weber, Mariano Esteban, Peter Liljeström

**Affiliations:** 1 Department of Microbiology, Tumor and Cell Biology, Karolinska Institutet, Stockholm, Sweden; 2 Imperial College London, Department of Infectious Diseases, Division of Medicine, Norfolk Place, London, United Kingdom; 3 Department of Molecular and Cellular Biology, Centro Nacional de Biotecnología, Consejo Superior de Investigaciones Científicas, Madrid, Spain; Mayo Clinic, UNITED STATES

## Abstract

Vaccination with DNA is an attractive strategy for induction of pathogen-specific T cells and antibodies. Studies in humans have shown that DNA vaccines are safe, but their immunogenicity needs further improvement. As a step towards this goal, we have previously demonstrated that immunogenicity is increased with the use of an alphavirus DNA-launched replicon (DREP) vector compared to conventional DNA vaccines. In this study, we investigated the effect of varying the dose and number of administrations of DREP when given as a prime prior to a heterologous boost with poxvirus vector (MVA) and/or HIV gp140 protein formulated in glucopyranosyl lipid A (GLA-AF) adjuvant. The DREP and MVA vaccine constructs encoded Env and a Gag-Pol-Nef fusion protein from HIV clade C. One to three administrations of 0.2 μg DREP induced lower HIV-specific T cell and IgG responses than the equivalent number of immunizations with 10 μg DREP. However, the two doses were equally efficient as a priming component in a heterologous prime-boost regimen. The magnitude of immune responses depended on the number of priming immunizations rather than the dose. A single low dose of DREP prior to a heterologous boost resulted in greatly increased immune responses compared to MVA or protein antigen alone, demonstrating that a mere 0.2 μg DREP was sufficient for priming immune responses. Following a DREP prime, T cell responses were expanded greatly by an MVA boost, and IgG responses were also expanded when boosted with protein antigen. When MVA and protein were administered simultaneously following multiple DREP primes, responses were slightly compromised compared to administering them sequentially. In conclusion, we have demonstrated efficient priming of HIV-specific T cell and IgG responses with a low dose of DREP, and shown that the priming effect depends on number of primes administered rather than dose.

## Introduction

An efficacious HIV vaccine will be required to induce strong and broad antibody and T cell responses [1]. To date, such responses have been difficult to obtain using single vaccine modalities, and attempts have been made to improve the immune response with heterologous prime-boost combinations, i.e. priming and boosting with different vaccine modalities. Such combinations have included priming with a DNA vaccine followed by boosting with a virus vector vaccine [2–9]. DNA vectors represent an attractive vaccine platform due to their ability to stimulate cellular and humoral immune responses. In human trials, DNA vaccines have been shown to be safe in thousands of volunteers. In particular, DNA vaccines have been shown to be excellent as a priming agent in prime-boost vaccine regimens [7, 9]. Also, improved delivery technologies such as *in vivo* electroporation (EP) greatly enhance the immunogenicity of DNA vaccines [4, 10, 11]. However, several studies have shown that achieving strong, broad and long-lasting immune responses have required repeated priming with high doses of DNA. Therefore, more work is needed to increase the immunogenicity of DNA vaccines for this platform to be viable for the development of future human vaccines.

We and others have previously shown that the immunogenicity of DNA vaccines can be increased with the use of DNA-launched alphavirus replicon vectors (DREP) [4, 12–16]. With this technology, the genes encoding the structural proteins of the alphavirus genome have been replaced with an immunogen of interest (S1 Fig.). When the alphavirus replicase is translated, it drives amplification of alphavirus RNA. This leads to the production of several RNA intermediates that stimulate pattern recognition receptors of innate immunity including Toll-like receptor (TLR) 3, TLR7, TLR8, melanoma differentiation-associated gene 5 product (MDA-5) and protein kinase R (PKR) [17–19]. This results in induction of a strong type I interferon (IFN) response, apoptosis and thereby promotion of cross-priming of antigen epitopes on MHC class I molecules [20–23]. Thus, the DREP vector carries intrinsic immunostimulatory properties, and induces stronger antigen-specific responses compared to conventional plasmid DNA vectors [4, 12–15, 24]. The use of intradermal (i.d.) EP for delivery of DREP enhances uptake and expression of the DNA, thus further increasing antigen-specific immune responses and allowing for a dose-sparing effect [4]. The DREP platform has been evaluated in a number of studies using a variety of model antigens and has proven to be a promising platform for generation of robust T and B cell immune responses [3, 4, 12–15, 24, 25].

We have previously demonstrated the induction of strong T cell responses against an HIV immunogen following a single prime immunization with a low dose of DREP and boosting with modified vaccinia virus Ankara (MVA) [4]. Similarly, in an experimental chikungunya virus vaccine, strong T cell as well as antibody responses were induced by boosting with both MVA and protein antigen simultaneously after a DREP prime [25]. Thus, while the poxvirus vector greatly boosts and expands T cell responses when administered after a DNA vaccine prime [2–9], a protein antigen formulated in adjuvant is optimal for inducing strong antigen-specific antibody responses [26, 27]. Therefore, a vaccination schedule encompassing a booster immunization with both a virus vector as well as a protein antigen boost would appear to be an excellent approach to induce antigen-specific responses from both arms of adaptive immunity [8, 25].

In the present study we evaluated the DREP platform in a murine model for the development of an HIV vaccine by inserting HIV genetic sequences that already are in clinical trials [8]. We evaluated the ability of DREP to generate robust immune responses on its own or in combination with recombinant MVA or in combination with trimeric recombinant gp140 HIV envelope protein formulated in an aqueous glucopyranosyl lipid A formulation (GLA-AF adjuvant), or in combination with MVA plus gp140 together. We investigated how dose and number of immunizations of DREP affects the immune response after a heterologous booster. Lower doses and shorter immunization regimens would be favorable in a clinical setting, as production costs could be reduced and compliance increased.

## Materials and Methods

### DNA and viral vectors, recombinant trimeric HIV-1 gp140 and GLA-AF

Two DNA vector backbones were used in these studies: A conventional plasmid DNA vector and a Semliki Forest virus DNA-launched replicon (DREP). For each vector backbone, two different constructs were used. One contained the HIV CN54 *Env* gene (DREP-C-ENV and DNA-C-ENV) and the other a HIV ZM96 *Gag-Pol-Nef* genetic fusion construct (DREP-C-GPN and DNA-C-GPN). For clarity, the terms DNA-C and DREP-C will be used when referring to both of the constructs with either conventional DNA or DREP backbone, respectively.

DNA-C has been described previously [8]. DREP constructs encoding HIV CN54 *Env* or HIV ZM96 *Gag-Pol-Nef* were constructed by first amplifying the *Env* and *Gag-Pol-Nef* genes from the conventional DNA-C plasmids. The primers contained overhangs encoding cleavage sites for *Xma*I and *Spe*I for amplification of *Env*, and *Afe*I and *Spe*I for amplification of *Gag-Pol-Nef*. The *Env* and *Gag-Pol-Nef* PCR products were then inserted into DREP-HIVconsv [4] that had been digested with either *Xma*I and *Spe*I (for *Env*), or *Sma*I and *Spe*I (for *Gag-Pol-Nef*). The correctness of the insert sequences were verified by sequencing.

The MVA pox vector expressing the CN54gp120 Env and Gag-Pol-Nef polyprotein from two back-to-back synthetic Early/Late transcriptional promoters (MVA-C) has been described previously [8, 28] and was manufactured by Bavarian Nordic (Kvistgaard, Denmark).

gp140 is a trimeric CN54 gp140 HIV clade C envelope (gp120 plus the external domain of gp41) manufactured by Polymun Scientific Immunbiologische Forschung GmbH (Vienna, Austria), and the TLR4 agonist GLA-AF a synthetic Monophosphoryl Lipid A in an aqueous formula manufactured by IDRI (Seattle, USA) that have been described previously [8, 29].

### Mice

Female BALB/c mice were bred either at the animal facility at the Department of Microbiology, Tumor and Cell Biology (MTC) at Karolinska Institutet, Sweden, or purchased from Scanbur (Sollentuna, Sweden). Animals were kept at MTC in accordance with the recommendations of the National Board for Laboratory Animals. Mice were 6–12 weeks old at the initiation of experiments. At termination of experiments, mice were sacrificed by cervical dislocation. The protocol was approved by the local ethics committee, Stockholms norra djurförsöksetiska nämnd, Permit Numbers N191/11 and N82/14.

### Immunizations and *in vivo* EP

Prior to immunization, mice were anesthetized with 2–3% isoflurane.

DNA-C, MVA-C, gp140 and GLA-AF were administered by intramuscular (i.m.) immunization. Mice were immunized 50 μl in one or both hind legs in the gastrocnemius muscle at doses indicated in the figure legends.

DREP-C-ENV and DREP-C-GPN were given with intradermal (i.d.) electroporation (EP), as described previously [4, 30]. Mice were immunized i.d. within a small shaved area at the base of the tail with a total of 40 μl into two sites (20 μl each) with a 30-gauge insulin-grade syringe (BD Micro-Fine; BD Consumer Healthcare, Franklin Lakes, NJ). Immediately after injection, a needle array electrode with two parallel rows of four 2-mm pins (1.5 × 4 mm gaps) was placed at the injected spot, and voltage was applied (2 pulses of 1,125 V/cm for 50 μs followed by 8 pulses of 275 V/cm for 10 ms). EP was performed using the DermaVax DNA vaccine skin delivery system (Cellectis, Paris, France).

### Isolation of splenocytes

To obtain single-cell suspensions of splenocytes, fresh mouse spleens were mashed through 70-μm cell strainers. Cells were then washed in complete RPMI medium (RPMI 1640 supplemented with 5% FCS, 2 mM L-glutamine, 100 U/ml penicillin and 100 μg/ml streptocymin [all from Gibco, Invitrogen]), treated with red blood cell lysis buffer (Sigma-Aldrich, St. Louis, MO) for 2 min, washed again, and resuspended in complete RPMI medium. Cells were quantified with the Countess automated cell counter (Invitrogen).

### IFN-γ ELISpot assay

MultiScreen-IP plates (Millipore, Billerica, MA) were activated with 70% ethanol, then washed four times with phosphate-buffered saline (PBS), and coated with anti-IFN-γ antibodies (AN18; Mabtech, Nacka Strand, Sweden) diluted in PBS overnight at 4°C. After five washes with PBS, plates were blocked with complete RPMI medium for ≥2h at 37°C. Blocking medium was then replaced by 2 × 10^5^ freshly isolated splenocytes per well in triplicates with either 2 μg/ml peptide, medium alone or 2 μg/ml of concavalin A (Sigma-Aldrich). Three different peptides were used in this study, derived from Env (PADPNPQEM) and Pol (VGPTPVNI and YYDPSKDLI) [31]. After 20±2h of incubation at 37°C with 5% CO_2_, plates were developed as recommended by the manufacturer with biotinylated anti-IFN-γ detector antibody (R4-6A2), streptavidin-alkaline phosphatase and BCIP-NBT Plus substrate (all Mabtech). Plates were analyzed using the ImmunoSpot analyzer and software (Cellular Technology Ltd., Bonn, Germany). Results are expressed as group means.

### Intracellular cytokine staining (ICS)

Freshly isolated splenocytes were kept in complete RPMI medium at 4°C and then set up in a 96-well round-bottom plate with 3 × 10^6^ splenocytes per well. Samples were incubated for 4h at 37°C with Golgi Stop, anti-CD107a-PE-Cy7 (1D4B; both BD Biosciences, San Diego, CA) and 2 μg/ml of each of the peptides PADPNPQEM, VGPTPVNI and YYDPSKDLI. For each sample, a medium control was incubated without peptides. Fc block (2.4G2; BD Biosciences) was added 10 min before the end of incubation. Cells were then washed with fluorescence-activated cell sorting (FACS) buffer (0.1% bovine serum albumin (BSA; Gibco, Invitrogen) in PBS), incubated with anti-CD8-Pacific Blue (53–6.7; BD Biosciences) in FACS buffer, and washed again. Cells were subsequently fixed and permeabilized with Cytofix/Cytoperm (BD Biosciences) and washed again. Staining was then performed with anti-interleukin-2 (IL-2)-APC (JES6-5H4), anti-IFN-γ-FITC (XMG1.2), and anti-tumor necrosis factor (TNF)-PE (MP6-XT22) (all from BD Biosciences) in Perm/Wash buffer (BD Biosciences). After washing in Perm/Wash buffer, cells were resuspended in FACS buffer. Samples were analyzed on a flow cytometer (FACSCanto II; BD Biosciences) followed by data analysis using the FlowJo software (Tree Star, Inc., Ashland, OR, USA). Background values from medium controls were subtracted from values of peptide-stimulated samples. Results are represented as group means.

### ELISA

ELISA plates (Immunosorp, Nunc, Denmark) were coated overnight with 1 μg/ml gp140 diluted in 0.1 M carbonate buffer at 4°C. Plates were then washed three times with PBS plus 0.05% Tween and blocked with 1% BSA for 1 h at room temperature. Subsequently, serum was serially diluted in PBS plus 0.05% Tween and incubated overnight at 4°C. After washing the plates three times with PBS plus 0.05% Tween, horseradish peroxidase-conjugated anti-mouse-IgG, anti-mouse-IgG1 or anti-mouse-IgG2a (all Southern Biotech, Birmingham, AL) was added and incubated for 1.5 h at room temperature. After five washes with PBS plus 0.05% Tween, the o-phenylenediamine dihydrochloride substrate (Sigma-Aldrich) was added for detection of antibodies. After 20 min, the reaction was stopped with 1 M HCl and the optical density (OD) at 490 nm was read using and ELISA reader. For calculation of endpoint titers, a cutoff value of OD 0.15 was used. Results are expressed as group means+SEM.

### Statistics

The GraphPad Prism 5 software (GraphPad Software Inc., La Jolla, CA, USA) was used for statistical analyses. For pairwise analysis between two groups, we used the nonparametric Mann-Whitney U test. For multiple comparisons, we performed the Kruskal-Wallis test for *a priori* analysis, followed by a *post hoc* analysis with Dunn’s test. A *P*-value of less than 0.05 was considered statistically significant. For statistical analysis of IFN-γ ELISpot data, we compared the total numbers of spots against all three epitopes between the different groups.

## Results

### DREP-C vaccine constructs

In this study, we constructed two DNA vaccines: DREP-C-ENV and DREP-C-GPN (collectively termed DREP-C). These constructs encode Env and a Gag-Pol-Nef fusion protein, respectively, both derived from an HIV-1 clade C strain. The constructs are based on DREP, a DNA-launched SFV replicon vector, which encodes the SFV replicase under control of a CMV promoter and the antigen genes under control of the SFV 26S subgenomic promoter (S1 Fig.). When cells are transfected with DREP, RNA is transcribed from DREP in the nucleus and transported to the cytoplasm where the SFV replicase is translated from the 5′ proximal portion of the RNA. The replicase then drives amplification of the DREP RNA as well as transcription of subgenomic RNA encoding the transgene, from which the transgene can be translated.

### DREP-C vaccine constructs are immunogenic in mice

We first confirmed that DREP-C constructs were immunogenic in mice. Mice were immunized with one of the constructs DREP-C-ENV or DREP-C-GPN, or with both of the constructs. Mice that were given both constructs were divided into two groups. One group was given a mix of the two plasmids divided equally in two injection sites on each side of the mouse. Another group was given DREP-C-ENV on one side and DREP-C-GPN on the other side. This was done to assess whether immunodominance would occur when mixing the two constructs, and if so, whether it perhaps could be circumvented by administering the constructs into two separate sites.

**Figure 1:**
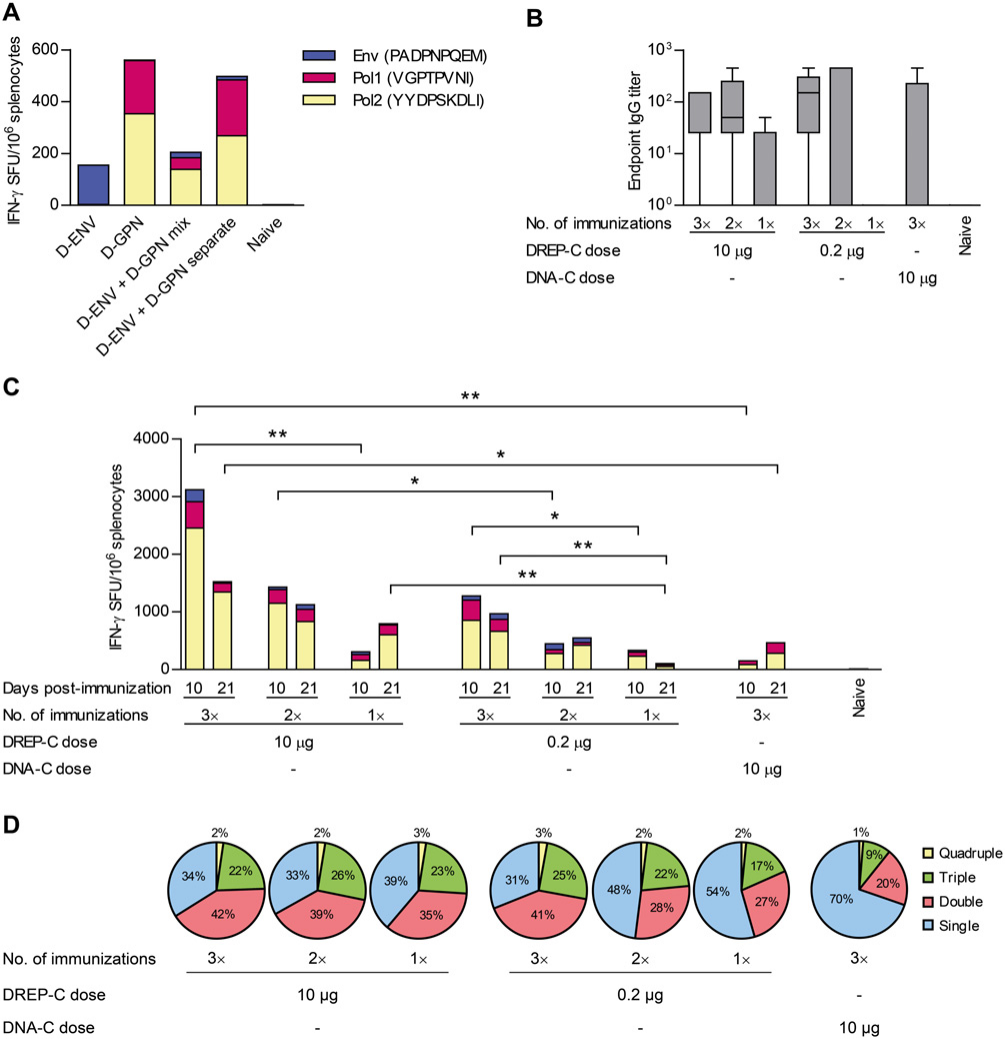
T cell responses induced by tested vaccine candidates. (A) Mice (*n* = 3 per group) were immunized once with 5 μg of DREP-C-ENV or 5 μg of DREP-C-GPN alone, or both constructs either mixed or given at separate sites. DREP constructs were given i.d. followed by EP. Splenocytes were assayed 10 days post-immunization with IFN-γ Elispot using peptides PADPNQEM (Env), VGPTPVNI (Pol1) and YYDPSKDLI (Pol2). (B-D) DREP-C-ENV and DREP-C-GPN induce T cells and antibodies that are boosted by multiple administrations. Mice (*n* = 5 per group) were immunized by i.d. EP one to three times with 5 or 0.1 μg of each DREP-C-ENV and DREP-C-GPN given at separate sites. One group was given DNA-C (5 μg of each construct) 3 times i.m. Boost immunizations were given with a 3 week interval between each administration. (B) Serum was assayed with ELISA for anti-gp140 IgG antibodies 3 weeks after the last immunization. Responses are shown as box plots, with whiskers representing 5–95 percentiles. (C) Splenocytes were assayed with IFN-γ Elispot 10 days and 3 weeks after last immunization using the peptides described above. (D) ICS for CD107a, IFN-γ, IL-2 and TNF-α was performed on splenocytes 10 days after the last immunization. The percentage of CD8+ T cells expressing a certain number of cytokines is shown. Specific markers expressed are shown in S2 Fig. Statistical analyses were performed to compare mice given a different number of administrations of the same dose of DREP-C. In addition, mice given the same number of administrations but with different doses/regimens were compared in separate statistical comparisons. Abbreviations: D-ENV, DREP-C-CN54ENV; D-GPN, DREP-C-ZM96GPN. * *P* < 0.05; ** *P* < 0.01

On day 10 after one single immunization, spleens were collected and assayed with IFN-γ ELISPOT for detection of antigen-specific T cells. A response against Env was observed in mice that were given only the DREP-C-ENV vaccine, and against both Pol1 and Pol2 in mice given only the DREP-C-GPN vaccine (Fig. 1A). Mice that were given a mixture of the two plasmids displayed a response against all three epitopes, although the responses were reduced to less than half compared to administering only one of the plasmids, demonstrating that each of the plasmids reduced the immune responses against the immunogen encoded by the other plasmid. Separating the two plasmids and administering them into two anatomically separate sites restored the responses against Pol1 and Pol2, although the response against Env was still substantially compromised compared to the response in mice given only DREP-C-ENV. Nevertheless, as the results showed that the magnitudes of the T cell responses were reduced when mixing the two plasmids compared to administering them separately, we chose the latter as the approach in all subsequent studies, for which the two plasmids will collectively be referred to as DREP-C. In addition, serum was assayed for detection of anti-Env antibodies with ELISA. No antibodies were detected in any groups (data not shown).

### DREP-C induces T cell and antibody responses that increase in magnitude with homologous boosts

We have previously shown that the antigen-specific T cell response induced by DREP in mice reaches a maximal magnitude with a dose in the range of 2–10 μg and that this response can be further increased with two homologous boosts [4]. Here, we assessed the T cell responses achieved by immunizing three times with either a 0.2 μg (suboptimal) or 10 μg (optimal) dose of DREP-C. Some mice were boosted homologously once or twice with three-week intervals with the same dose to compare the response after one, two and three immunizations. In addition, one group of mice was given 10 μg of DNA-C encoding the same immunogens three times i.m. without EP. This was done to bridge our results to a previous study by McKay *et al*. [8]. The difference in administration is expected to contribute to the observed differences in immunogenicity; however, DREP is known to induce significantly stronger T cell responses than conventional DNA also when both modalities are administered with EP [4]. The anti-Env antibody response was analyzed at 3 weeks post-immunization by ELISA (Fig. 1B). A low but clearly detectable anti-Env IgG response was observed in all groups except in mice given one immunization of the low dose of DREP-C (Fig. 1B). Similar levels of anti-Env antibodies were observed after two and three immunizations of either dose of DREP-C or 3 immunizations of DNA-C. The responses were slightly lower in the group given one immunization of the high dose of DREP-C.

The T cell response induced by DREP peaks 10 days after a single immunization, and 8–10 days following a second immunization. We observed that the response had contracted substantially 2 weeks after the last immunization [3]. Therefore, T cell responses were assessed 10 days and 3 weeks after the last immunization with IFN-γ ELISPOT (Fig. 1C) as well as ICS on day 10 (Fig. 1D).

As expected, the magnitude of the T cell response increased with each homologous boost of DREP-C (Fig. 1C). This was evident both with the low and high dose and at both time points. Three immunizations of the low dose induced a response similar in magnitude to two immunizations of the higher dose. The response induced by three immunizations of DNA-C was lower than in any groups immunized with DREP-C on day 10, and at 3 weeks only higher than in mice given one immunization with the low dose of DREP-C.

Oligofunctional HIV-1-specific CD8+ T cells that produce multiple cytokines and have cytotoxic function have been associated with control of chronic HIV-1 infection [32–35]. Therefore, we assessed the ability of antigen-specific CD8+ T cells to produce effector cytokines IFN-γ, IL-2 and TNF-α as well as their cytotoxic function by detecting mobilization of degranulation marker CD107a. In groups given 10 μg of DREP-C, the distribution of CD8+ T cells that were single, double, triple or quadruple producers was similar after one, two or three immunizations (Fig. 1D). The distribution was similar in mice that were given three immunizations of 0.2 μg DREP-C; however, the proportion of cells that produced only one of the molecules was larger following two immunizations of 0.2 μg DREP-C, and even larger after only one immunization. Immunizing three times with 10 μg DNA-C resulted in a proportion of T cells producing only one marker that was substantially larger than in any of the groups given DREP-C. This proportion was in mice given DNA-C 2-fold larger than that observed after one to three immunizations of 10 μg DREP-C or three immunizations of 0.2 μg DREP-C. Overall, following the different immunization regimens, the phenotypes of triple-producers were mainly CD107a+IFN-γ+TNF-α+ and IFN-γ+IL-2+TNF-α+. Double-producers were mainly CD107a+IFN-γ+ and IFN-γ+TNF-α+, and single-producers were mainly IFN-γ+, although interestingly single-producers in mice given DNA-C were of both IFN-γ+ and TNF-α+ phenotypes (S2 Fig.). In a previous study, we observed similar proportions of oligofunctional T cells when an MVA boost was given following a DREP or conventional DNA prime [4].

### Heterologous prime-boost experimental setup

DREP is superior to conventional plasmid DNA as a priming component before a heterologous boost. Also, one prime immunization with only 50 ng DREP resulted in a response equivalent to that induced by priming with 2.5 μg of DREP [4]. Here we assessed the effect of administering different numbers of prime immunizations with DREP-C on the ability to prime antigen-specific T cell and antibody responses prior to a heterologous boost.

**Figure 2:**
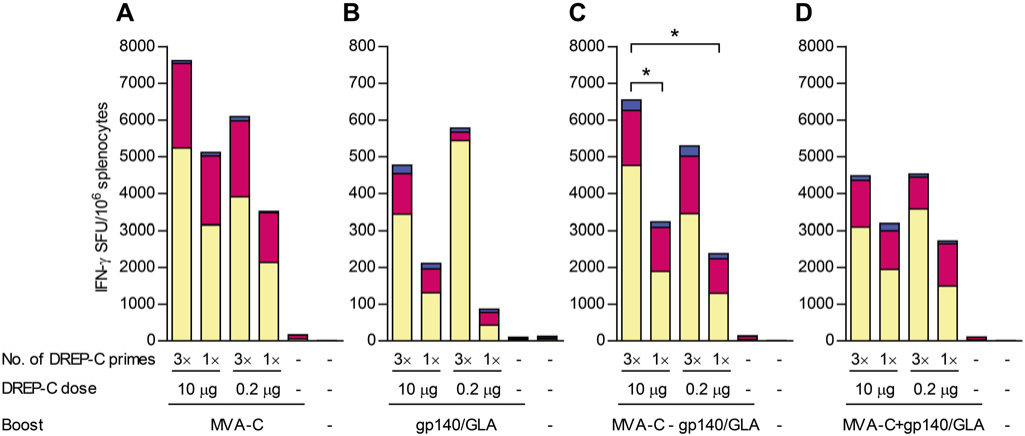
T cell responses following DREP-C prime and boost with MVA-C and/or gp140/GLA. Mice (*n* = 5 per group) were primed one or three times with DREP-C, as described in Fig. 1B-D. Six weeks after the last administration of DREP-C, mice were boosted with one of the following: (A) MVA in one side, (B) CN54gp140/GLA in one side, (C) MVA-C in one side followed by gp140/GLA in the other side three weeks after MVA-C administration, or (D) MVA-C in one side and gp140/GLA in the other side simultaneously. The DREP-C doses are stated in the figure. The booster doses given were 5×10^6^ TCID50 of MVA, 10 μg of gp140 mixed with 0.7 μg of GLA. Three weeks after the last immunization, splenocytes were assayed with IFN-γ Elispot. For peptide designation, see Fig. 1A. Groups that were given the same booster following a DREP-C prime were compared statistically. Please note that the Y-axis scale of (B) differs from the others. * *P* < 0.05.

**Figure 3:**
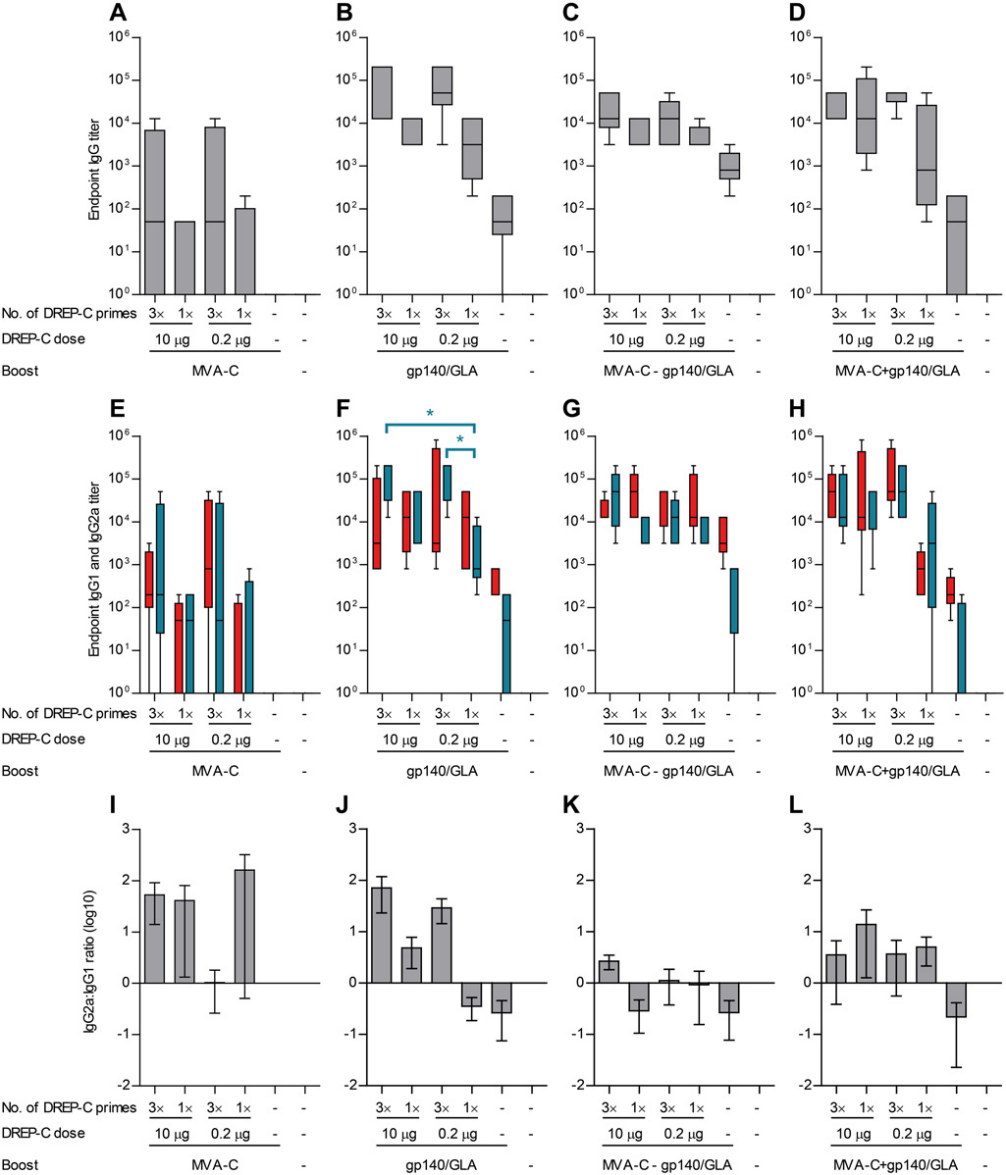
Antibody responses following DREP-C prime and boost with MVA-C and/or gp140/GLA. Mice (*n* = 5 per group) were primed 1 or 3 times with DREP-C, as described in Fig. 1B-D. Six weeks after the last administration of DREP-C, mice were boosted with one of the following: (A, E) MVA in one side, (B, F) CN54gp140/GLA in one side, (C, G) MVA-C in one side followed by CN54gp140/GLA in the other side three weeks after MVA-C administration, or (D, H) MVA-C in one side and gp140/GLA in the other side simultaneously. The DREP-C doses are stated in the figure. The booster doses given were 5×10^6^ TCID50 of MVA, 10 μg of gp140 mixed with 0.7 μg of GLA. Three weeks after the last immunization, serum was assayed with ELISA for anti-gp140 (A-D) IgG, (E-H) IgG1 (red) and IgG2a (blue). Responses are shown as box plots, with whiskers representing 5–95 percentiles. (I-L) IgG2a:IgG1 ratio means, with error bars representing standard error of the mean. Groups that were given the same booster following a DREP-C prime were compared statistically. * *P* < 0.05.

Mice were primed with either one or three immunizations of DREP-C, at a dose of either 0.2 μg or 10 μg, followed by boosting with either MVA-C, or gp140 formulated in GLA-AF adjuvant. The GLA-AF adjuvant has previously been shown to potentiate anti-Env antibody responses when co-administered with gp140 protein [8, 36, 37]. Multiple DREP-C immunizations were given with three-week intervals. Heterologous boost immunizations were given six weeks after the last immunization with DREP-C. In the experiments described below, gp140 was in all cases administered with GLA-AF, although for simplicity only gp140 will be stated. Furthermore, some mice were given both MVA-C and gp140, either at the same time in separate legs, or MVA-C as a first heterologous boost, followed by a second boost with gp140. Three weeks after the last immunization, splenocytes were assayed with IFN-γ ELISPOT for detection of T cell responses (Fig. 2), and serum was assayed with ELISA for anti-Env antibodies (Fig. 3).

### A single low dose of DREP-C immunization potently primes T cells that are expanded by an MVA boost

In mice that were given a boost with only MVA-C, the highest T cell response was induced in the group that was primed three times with the high dose of DREP-C (Fig. 2A). The response was lower although also high in mice primed three times with the low dose of DREP-C. The response was still lower in mice primed once with the high dose of DREP-C, and even lower in mice given the low dose of DREP-C once. However, all groups primed with DREP-C displayed a strong T cell response and did not differ significantly. Boosting with MVA-C after one DREP-C prime expanded the response 7-fold (low DREP-C dose) and 4-fold (high DREP-C dose) more than administering a homologous DREP-C boost (Fig. 1C). The response in mice that were given only MVA-C with no prime was 5-fold lower than the response induced by only one immunization of the high dose of DREP-C (Fig. 1C).

T cell responses were in the range of 10-fold lower in mice given a boost with CN54gp140 after a DREP-C prime, compared to after an MVA-C boost (Fig. 2B). The gp140 did not by itself induce a T cell response, and thus the responses observed in DREP-C-primed groups represented the response 9 weeks after immunization with a T cell stimulant and had consequently further contracted from the levels found at 3 weeks post immunization (Fig. 1C). Again, the highest responses were seen in mice primed three times with a low or high dose of DREP-C. The response was lower in mice given a single prime immunization of either the low or high dose of DREP-C, although higher in both groups than in mice given only gp140 without a DREP-C prime in which a T cell response was undetectable.

Since MVA-C potentiated T cell responses while protein antigen induces strong antibody responses, we assessed whether we could obtain both a strong T cell and antibody response by boosting with both MVA-C and gp140. We therefore boosted mice sequentially with first MVA-C followed by a boost with gp140. T cell responses were overall slightly lower but similar to the responses induced by MVA-C alone (Fig. 2C). Since the experiments were designed in such a manner that the responses were analyzed at the same time point after the last immunization, the analyses occurred at a later time after the MVA-C boost in mice that were also given gp140 (6 weeks) compared to those that were given only a MVA-C boost (3 weeks). Responses were, however, still at a high level, illustrating slow contraction between 3 and 6 weeks post-boost, which is consistent with a powerful boost. Comparing the different DREP-C prime regimens, we again observed the strongest responses in mice that were given three immunizations of a high or low dose of DREP-C. Responses in mice that were given only a single administration of DREP-C prior to boost displayed a lower response, although significantly higher than in mice given MVA-C and gp140 without a DREP-C prime, for which T cell responses were undetectable against Env and Pol2 epitopes, and close to undetectable against epitope Pol1.

Next we asked whether we could shorten the vaccine regimen by administering MVA-C and gp140 simultaneously. In mice given both MVA-C and gp140 at the same time (Fig. 2D), the magnitude of the T cell responses were overall slightly reduced compared to after a boost with only MVA-C, indicating that co-administration of protein antigen inhibits virus-induced T cell responses. Responses were also slightly lower than in mice given the sequential MVA-C and gp140 boost, especially in mice given three prime immunizations. Again, the strongest responses were observed when three doses of DREP-C had been given prior to the heterologous boost, regardless of the dose of DREP-C. All groups primed with DREP-C had a significantly stronger T cell response than mice given only MVA-C and gp140 without a prime, which displayed only a low response against epitope Pol1, demonstrating that even a single prime with only 0.2 μg of DREP-C has a significant impact on formation of memory T cells that can be boosted to high levels.

### A single low dose of DREP-C immunization primes anti-gp140 IgG responses that are increased by a gp140 protein boost

Anti-gp140 IgG levels following an MVA-C boost were similar in mice primed an equal number of times with DREP-C, regardless of dose (Fig. 3A). Mice primed three times with the high or low dose of DREP-C displayed a stronger antibody response than mice primed only once. IgG1 and IgG2a subtype levels were similar between groups primed an equal number of times (Fig. 3E and I). An antibody response was not induced in the non-primed group that was immunized only with MVA-C, demonstrating that DREP-C alone (Fig. 1B) was more potent at inducing antibodies than MVA-C. Thus, administering DREP-C as a prime prior to an MVA-C boost resulted in induction of antibodies.

The antibody responses were substantially higher in mice given the gp140 boost compared to those given the MVA-C boost (Fig. 3B). The response was strong in all groups given a DREP-C prime, although higher in groups given three prime immunizations compared to only one prime immunization. Again, the response depended on the number of DREP-C prime immunizations rather than the dose. A low but detectable response was induced in mice given only gp140 without a DREP-C prime, at a similar level as that induced by two or three immunizations of the high or low dose of DREP-C (Fig. 1B). Mice given three DREP-C prime immunizations displayed a higher IgG2a:IgG1 ratio compared to mice given only one prime immunization, indicating that the multiple administrations of DREP-C favored development of a Th1 response (Fig. 3F and J). Mice given only gp140 without a prime had a stronger IgG1 response compared the IgG2a response in these mice, demonstrating that protein/GLA-AF skews the response to a Th2 phenotype.

Anti-gp140 IgG levels were similar in mice given three prime immunizations of high or low dose of DREP-C prior to the sequential MVA-C and gp140 boost (Fig. 3C). Levels were slightly lower in mice primed once with DREP-C, and even lower in mice given only MVA-C and gp140 without a DREP-C prime. Compared to giving only a gp140 boost, the response was slightly lower in the mice given three DREP-C prime immunizations. IgG2a were slightly elevated and IgG1 levels lowered in mice given three DREP-C prime immunizations compared to only one prime immunization, indicating that the multiple administrations of DREP-C favors development of a Th1 response (Fig. 3G and K).

After boosting with MVA-C and gp140 simultaneously, anti-gp140 IgG levels were highest in the groups given three prime immunizations with a high or low dose of DREP-C as well as in mice given a single administration of a high dose of DREP-C (Fig. 3D, H, L). IgG levels were slightly lower in the group given one immunization of the low dose of DREP-C, although still substantially higher than the group not primed with DREP-C. This non-primed group displayed an IgG response lower than in mice given MVA-C and gp140 sequentially rather than simultaneously (Fig. 3C), demonstrating that MVA-C negatively impacts induction of IgG against a protein antigen when given at the same time.

## Discussion

In the present study, we evaluated antigen-specific T cell and antibody responses following immunization with different doses and numbers of immunizations of DREP-C, and asked what effect changing these variables would have when given as a prime immunization prior to a heterologous boost. Although one to three administrations of 0.2 μg of DREP-C by itself induced lower responses than the equivalent number of immunizations of 10 μg DREP-C, the two doses primed T cell and antibody responses similarly, resulting in comparable magnitudes of response after a heterologous boost. This suggests that dose sparing can be obtained with the use of DREP rather than conventional DNA, as the doses of DNA commonly used are up to 500-fold higher than the low dose evaluated in this study [8]. Also, we have previously demonstrated that antigen-specific CD8+ T cell responses remain substantial five weeks after immunization with DREP and are characterized by both effector and central memory cells [3, 38].

Immunodominance, i.e. a response directed against only one or a few dominant epitopes out of many possible epitopes, is known to occur with HIV antigens [39, 40]. Not surprisingly, we observed here that a DREP approach did not circumvent such an immunodominance phenomenon. Previous studies have shown that immunodominant immune responses can be avoided by administering the different DNA vaccine-vectored immunogens into anatomically separate sites [41, 42]. Therefore, we administered DREP-C-ENV and DREP-C-GPN into separate sites. With this approach, the Pol-specific T cell responses were restored compared to the response after administering the two plasmids mixed. However, Env-specific T cell responses were reduced compared to administering DREP-C-ENV into the two sites without DREP-C-GPN. The reason for this could be that Env is a weaker epitope than the Pol epitopes, and that the Env-specific response is therefore reduced when it is only administered in one anatomical location compared to multiples sites as was the case when DREP-C-ENV was tested alone. Moreover, it is unclear how anatomically distinct the injection sites actually are. It is possible that, although we immunize on different sides of the spine on the lower back of the mouse, immune induction occurs at least partially in the same draining lymph node.

The role of T cell polyfunctionality on HIV disease progression is of central importance. Polyfunctional T cells have been shown to be central in protection against disease; however, their exact role has not been entirely clarified [32–35]. Recently it was shown that it probably is the strength of the polyfunctional T cell response that is critical to control HIV disease progression and that increased diversity or specific key functions of polyfunctional T cells play a lesser role [43]. We show here that DREP-primed immune responses appear to have broad polyfunctionality. When immunizing with a suboptimal dose of DREP-C, polyfunctionality increased during secondary and tertiary responses.

Priming with a single shot of either 0.2 μg or 10 μg of DREP-C prior to a heterologous boost did not induce equally high responses as priming with DREP-C three times, indicating that the number of priming immunizations affects the outcome of the response more than the dose used. However, one priming immunization with DREP did increase antigen-specific T cell and antibody responses substantially. For example, T cell responses were increased 20-fold by priming once with 0.2 μg of DREP-C prior to an MVA-C boost compared to immunizing with MVA-C only. Administering three prime immunizations further increased the response 1.6-fold compared to priming only once. Thus, the largest impact on the magnitude of the response was obtained by administering at least one prime with DREP-C. Although the response can be further increased by multiple administrations of DREP-C, decreasing the dosage of the vaccine regimen to only one prime immunization has advantages in a clinical setting such as decreased costs and shortened vaccine schedule that may have benefits for compliance.

We observed that priming with DREP-C resulted in potent antibody responses of both IgG1 and IgG2a isotypes, whereas protein/adjuvant induced mainly an IgG1 response that was not affected by MVA-C. IgG1 is mainly associated with a Th2 type response, whereas IgG2a is associated with a Th1 type response with the ability to mediate antibody-dependent cellular cytotoxicity and complement activation [44, 45]. Non-neutralizing antibodies with these properties have recently been suggested to have a key role against HIV infection [46].

Although not completely understood, the high immunogenicity of the DREP vector is most likely based on its ability to stimulate multiple PRRs, leading to induction of robust type I IFN response as well as apoptosis [17–19, 21–23]. Co-delivery of alphavirus replicons as viral particles (VREP) with protein antigen potentiates IgG responses against the antigen [47, 48]. This adjuvant activity is dependent on type I IFN signaling. However, type I IFNs suppress the antigen-specific CD8+ T cell response induced by high doses of DREP or by alphavirus replicon viral particles [3, 4, 24]. This is likely due to the antiviral state induced by type IFNs, inhibiting production and amplification of replicon RNA. Also, DREP-induced T cell responses are not significantly compromised in mice lacking TLR3, TLR7 or TLR9 [24].

In this study we also assessed the effect of boosting with both poxvirus-vectored antigen and protein antigen formulated in GLA adjuvant following DREP-C priming. Since MVA-C boosted T cells, and gp140 primarily boosted antibodies, we asked if we could boost both T cells and antibodies by administering both vaccine components. A shorter vaccine schedule would be favorable in a clinical setting, as it would lead to increased compliance. Therefore, we asked whether there would be a difference in the boosting effect if we administered MVA-C and gp140 simultaneously as compared to giving them sequentially. We did not observe major differences if the boosts were given sequentially or simultaneously, suggesting that the length of the vaccine regimen can be shortened. Administration of gp140 together with MVA, however, decreased T cell responses induced by MVA. A recent study used the same MVA-C and gp140 boosts as in the present study after a non-replicon DNA plasmid prime administered i.m. without EP [8]. In that study, humoral responses were also similar when giving the two boosts simultaneously rather than sequentially. However, T cell responses were enhanced after priming with conventional DNA when administering the two boosts at the same time, and not at a slightly lower level as observed in the present study. The increased T cell response in that study may have been due to the higher dose of GLA adjuvant exerting a systemic immunostimulatory effect that enhanced responses against immunogens encoded by MVA-C.

An additional aspect that could be considered is whether there would be a difference in reversing the order of boosting with MVA-C and gp140/GLA in the sequential vaccine schedule. One study showed that priming with gp140 with IC31 adjuvant followed by boosting with another poxvirus vector, New York vaccinia virus (NYVAC), induced Env-specific CD4+ and CD8+ T cell responses that were superior to those induced by the reverse order of protein and NYVAC administration [49]. In this study, sequential administration of MVA-C and gp140/GLA did not induce a T cell response against Env. This vaccine regimen in part mimicked the recent Thai HIV vaccine trial RV144, which was a prime-boost vaccination schedule using recombinant canary poxvirus (ALVAC-HIV) followed by two gp120 protein boosts (AIDSVAX B/E) [50].

In planning prime-boost regimens, a different strategy could to induce T cells and antibodies separately rather than simultaneously, in order to ensure sufficient induction of both arms. We observed here that DNA prime-protein boost was sufficient for induction of antibodies, whereas DNA prime-MVA boost potently induced T cells. Also, the different immunogens could be delivered at different time points in order to avoid immunodominance. For example, a DNA prime-protein boost vaccine regimen using an optimized DNA vector encoding HIV gp140 and recombinant gp120 protein induced Env-specific T and B cells as well as neutralizing antibodies [51].

In conclusion, we demonstrate that the use of DREP as the prime component in a heterologous prime-boost regimen required only 0.2 μg per immunization. Compared to a recent preclinical study using identical boost components [8], this is a 500-fold reduction in dose, suggesting that a dose-sparing effect can be achieved by the replacement of conventional plasmid DNA with DREP. These results, in addition to our previous study demonstrating that only nanogram quantities of DREP are required for induction of CD8+ T cell responses [4], strongly suggest a replacement of DREP as the priming component in clinical heterologous prime-boost vaccines.

## Supporting Information

S1 Fig Alphavirus replicon amplification cycle. After transfection, DREP is transcribed into replicon RNA, from which the alphavirus replicase is translated. The replicase then drives amplification of replicon RNA as well as transcription of subgenomic RNA encoding an immunogen of interest. From this RNA, large amounts of immunogen are produced.
(EPS)

S2 Fig ICS for CD107a, IFN-γ, IL-2 and TNF-α was performed on splenocytes 10 days after the last immunization. Specific markers expressed are shown.
(EPS)
